# Statin-Associated Necrotizing Autoimmune Myopathy: Unmasking 3-Hydroxy-3-Methylglutaryl-CoA Reductase (HMGCR) Antibody-Positive Disease After Statin Re-exposure

**DOI:** 10.7759/cureus.89275

**Published:** 2025-08-03

**Authors:** Aurora Cafuli, Anthony Martins, Nosagie Ohonba, Donahue K Johnson, Sofia Terner

**Affiliations:** 1 Internal Medicine, Overlook Medical Center, Summit, USA; 2 Internal Medicine, Ocean University Medical Center, Brick, USA

**Keywords:** anti-hmgcr antibodies, creatine kinase elevation, immune-mediated myositis, immunosuppressive therapy, necrotizing autoimmune myopathy, proximal muscle weakness, statin-induced autoimmune necrotizing myopathy, statin-induced myopathy, statin reinitiation, steroid-sparing agents

## Abstract

Necrotizing autoimmune myopathy (NAM) is an uncommon inflammatory muscle disease marked by progressive weakness and elevated muscle enzymes. In some individuals, it may develop in association with statin use, particularly when specific autoantibodies are present. We report the case of a 65-year-old man who experienced worsening, painless proximal muscle weakness and significant creatine kinase (CK) elevation shortly after resuming statin therapy following a temporary discontinuation. The absence of myalgias or systemic symptoms obscured the diagnosis, and initial elevations in liver enzymes were misattributed to known hepatic steatosis during outpatient evaluation. Diagnostic workup revealed positive 3-hydroxy-3-methylglutaryl-CoA reductase antibodies (HMGCR Ab), magnetic resonance imaging (MRI) findings consistent with inflammatory myopathy, and a muscle biopsy demonstrating scattered necrotic and regenerating fibers, characteristic of immune-mediated necrotizing myopathy (IMNM). This case highlights the potential for statin re-exposure to unmask or exacerbate evolving NAM in patients with subclinical disease. Clinicians should maintain a high index of suspicion for NAM in statin-treated individuals presenting with unexplained muscle weakness or transaminitis. Early recognition and timely initiation of therapy are critical to prevent irreversible muscle damage and improve clinical outcomes.

## Introduction

Statins are widely used medications for reducing cardiovascular risk and preventing heart attacks and strokes. While generally well tolerated, they can cause muscle-related side effects that vary in severity, from mild aches to rare autoimmune muscle diseases. One such condition, called statin-associated immune-mediated necrotizing myopathy (IMNM), often presents with progressive proximal muscle weakness, elevated creatine kinase (CK) levels, and specific autoantibodies against 3-hydroxy-3-methylglutaryl-CoA reductase antibodies (HMGCR Ab).

While self-limited statin-associated myalgias occur in up to 10% of users, IMNM remains rare, with an estimated incidence of two to three cases per 100,000 statin users [1]. Importantly, unlike benign statin-induced myopathy, IMNM is an immune-mediated process that may progress despite statin discontinuation and typically requires corticosteroids and additional immunosuppressive therapy [1,2].

Emerging data suggest that prior exposure to statins may sensitize the immune system - a phenomenon referred to as immune priming - thereby allowing re-exposure to precipitate or exacerbate autoimmune muscle injury. This implies that patients previously tolerant of statins may later develop overt disease upon reinitiation, possibly due to upregulated HMGCR expression in regenerating muscle fibers and enhanced memory T-cell responses [1].

In this report, we describe a case of HMGCR ab-positive IMNM that worsened and became clinically apparent shortly after resuming statin therapy following a temporary discontinuation. The patient's lack of myalgias or systemic symptoms, along with the presence of transaminitis initially attributed to hepatic steatosis, obscured the diagnosis during early outpatient evaluation. This case highlights the importance of maintaining a high index of suspicion, recognizing that elevated liver enzymes may reflect skeletal muscle injury, and employing a multidisciplinary, structured diagnostic workup when evaluating statin-treated patients with unexplained proximal muscle weakness.

## Case presentation

A 65-year-old male with a past medical history of coronary artery disease (CAD), hypertension (HTN), hyperlipidemia (HLD), and type 2 diabetes mellitus (T2DM) was referred to the emergency department (ED) for evaluation of a markedly elevated serum CK level noted on outpatient labs. The patient had been undergoing follow-up with a gastroenterologist for suspected hepatic steatosis, during which routine laboratory monitoring revealed rising liver function tests (LFTs).

Approximately six weeks prior to admission, the patient’s outpatient laboratory workup revealed rising transaminases. As a result, his atorvastatin (40 mg nightly) and ezetimibe (10 mg daily) were discontinued. Subsequent evaluation, including viral hepatitis panel, autoimmune markers (ANA, ASMA, AMA, anti-LKM), iron studies, ceruloplasmin, and alpha-1 antitrypsin, was unremarkable. Abdominal ultrasound demonstrated moderate-to-severe hepatic steatosis without fibrosis, which was further confirmed by transient elastography (FibroScan, Echosens, Paris, France) showing a controlled attenuation parameter (CAP) of 291 dB/m and liver stiffness of 3.6 kPa.

Three weeks prior to admission, the patient began noticing subtle, progressive muscle weakness, initially affecting fine motor tasks such as clipping his phone to his waistband. Over the following week, this weakness progressed to involve the proximal muscles of both upper and lower extremities, manifesting as difficulty raising his arms above his head and rising from a seated position.

Two weeks prior to admission, the patient independently resumed atorvastatin and ezetimibe, approximately four weeks after their initial discontinuation, under the belief that his muscle weakness was unrelated to these medications. On the day of hospital admission, repeat outpatient follow-up labs revealed significantly elevated transaminases and a CK level, prompting referral to the ED and subsequent admission.

At presentation, he reported progressive, painless, symmetric proximal muscle weakness. He denied muscle pain, systemic symptoms, recent illness, trauma, alcohol use, supplement intake, medication changes (besides the reintroduction of statin and ezetimibe), rash, joint pain, oral ulcers, or weight loss. He also denied recent strenuous activity.

Vital signs were notable for HTN (180/107 mmHg) but were otherwise within normal limits. Physical examination revealed symmetric 4/5 strength in the deltoids, hip flexors, and neck flexors, with preserved distal strength, normal reflexes, and intact sensation. There were no upper motor neuron signs, cutaneous stigmata of dermatomyositis (e.g., heliotrope rash, Gottron’s papules), or evidence of synovitis. Cardiopulmonary and abdominal examinations were unremarkable.

Initial laboratory evaluation was significant for CK 15,767 U/L, aspartate aminotransferase (AST) 309 U/L, alanine aminotransferase (ALT) 293 U/L, and preserved renal function. Urinalysis demonstrated moderate blood without red blood cells, suggestive of myoglobinuria. Inflammatory markers were within normal limits (CRP <3 mg/L, ESR 18 mm/hour), TSH was normal, hemoglobin A1c was 7.6%, and a toxicology screen was negative. A complete summary of admission laboratory data is provided in Table 1. The patient was admitted with concern for necrotizing autoimmune myopathy (NAM), particularly the statin-associated, given the clinical context and lack of improvement following statin discontinuation.

**Table 1:** Laboratory Values Admission and Discharge ALT = alanine aminotransferase; AST = aspartate aminotransferase; BUN = blood urea nitrogen; CK = creatine kinase; CRP = C-reactive protein; ESR = erythrocyte sedimentation rate; HbA1c = hemoglobin A1c; HPF = high power field; RBC = red blood cells; TSH = thyroid-stimulating hormone; WBC = white blood cells *Tested for Phencyclidine, Benzodiazepines, Cocaine, Amphetamines, Cannabinoids, Opiates, Barbiturates, Methadone.

Lab Test	On Admission	On Discharge	Reference Range
Serum Glucose	159 mg/dL	182 mg/dL	70-100 mg/dL
BUN	24 mg/dL	16 mg/dL	7-18 mg/dL
Creatinine	0.64 mg/dL	0.61 mg/dL	0.0-1.3 mg/dL
Creatinine Kinase	15,767 U/L	9,110 U/L	20-200 U/L
AST	309 U/L	191 U/L	15-37 U/L
ALT	293 U/L	258 U/L	12-59 U/L
Creatinine Kinase	15,767 U/L	9,110 U/L	30-200 U/L
TSH	2.680 uIU/mL	Not Repeated	0.340-4.820 uIU/mL
ESR	18 mm/hour	Not Repeated	<30 mm/hour
CRP	<3.0 mg/L	Not Repeated	0-10 mg/L
A1c	7.6%	Not Repeated	4.0-5.6 %
Vitamin B12	747 pg/mL	Not Repeated	200-900 pg/mL
Folic Acid	9.8 ng/mL	Not Repeated	>5.4 ng/mL
Vitamin D 25-hydroxy	22.7 ng/mL	Not Repeated	30-100 ng/mL
Fasting Cortisol	9.4 mcg/dL	Not Repeated	5-25 mcg/dL
Alcohol level	<10.1 mg/dL	Not Repeated	<10.1 mg/dL
Toxicology Screen*	Negative	Not Repeated	Negative
Urine Color	Yellow	Not Repeated	Yellow-Amber
Appearance, Urine	Clear	Not Repeated	Clear
Glucose, Urine	Trace	Not Repeated	Negative
Ketones, Urine	Negative	Not Repeated	Negative
Bilirubin, Urine	Negative	Not Repeated	Negative
Specific Gravity	1.020	Not Repeated	1.005-1.030
Blood, Urine	Moderate	Not Repeated	Negative
pH, Urine	5.5	Not Repeated	5.0-8.0
Protein Urine, Total	Trace	Not Repeated	Negative
Urobilinogen, Urine	≤1.0 mg/dL	Not Repeated	≤1.0 mg/dL
Nitrite	Negative	Not Repeated	Negative
Leukocyte Esterase	Negative	Not Repeated	Negative
WBC, Urine	0-3/HPF	Not Repeated	0-3/HPF
RBC, Urine	0-3/HPF	Not Repeated	0-3/HPF
Squamous Epithelial	0-5/HPF	Not Repeated	0-5/HPF
Cast, Hyaline	0-5/HPF	Not Repeated	0-3/HPF
Bacteria, Urine	Not Present/HPF	Not Repeated	Not Present/HPF

The patient’s statin and ezetimibe therapy were discontinued on admission, and he received intravenous isotonic fluids throughout hospitalization to mitigate the risk of rhabdomyolysis-induced acute kidney injury. On admission day (day 0) and hospital day 1, he received normal saline (NS) at 125 cc/hour. The rate was increased to 150 cc/hour on day 2 and to 175 cc/hour on day 3 in response to persistently elevated CK levels. On day 4, the infusion was reduced to 150 cc/hour, followed by tapering to 100 cc/hour on day 5 and 75 cc/hour on day 6, as despite this volume support, CK levels remained elevated, plateauing near 11,000 U/L, raising further concern for underlying inflammatory myopathy rather than isolated rhabdomyolysis. Transaminases gradually trended downward over the hospital course. A myositis workup was sent that later revealed aldolase 119.8 U/L, serum myoglobin 2,220 ng/mL, and a positive HMGCR Ab titer of 160 U (reference <20 U). Myomarker panel was negative, ANA was negative, and other autoantibodies and infectious workup were unrevealing. See Table 2 for a complete list.

**Table 2:** Comprehensive Laboratory and Myomarker Results ANA = antinuclear antibody; Anti-PM/Scl-100 Ab = anti-polymyositis/scleroderma 100 antibody; Anti-SS-A 52kD Ab, IgG = anti-Sjögren's syndrome antigen A 52kD antibody, IgG; Anti-U1-RNP Ab = anti-U1 ribonucleoprotein antibody; EJ = glycyl-tRNA synthetase; Fibrillarin (U3 RNP) = fibrillarin antigen, a component of U3 ribonucleoprotein; HIV = human immunodeficiency virus; HMGCR Ab = 3-hydroxy-3-methylglutaryl-CoA reductase antibody; Ig = immunoglobulin; Ku = Ku autoantigen (DNA repair protein complex); MDA-5 (P140-CADM-140) = melanoma differentiation - associated gene 5; MI-2 = nucleosome remodeling deacetylase complex; NXP-2 (P140) = nuclear matrix protein 2; OJ = isoleucyl-tRNA synthetase; PL-12 = alanyl-tRNA synthetase; PL-7 = threonyl-tRNA synthetase; SRP = signal recognition particle; TB = tuberculosis; TIF1-γ (gamma, P155/140) = transcription intermediary factor 1 gamma; U2 snRNP = U2 small nuclear ribonucleoprotein

Test	Result	Reference Range/Interpretation
Serum IgG	1,077 mg/dL	700-1,600 mg/dL
Serum IgA	208 mg/dL	70-400 mg/dL
Serum IgM	62 mg/dL	40-230 mg/dL
QuantiFERON-TB Gold	Negative	Negative
HMGCR Ab	160 U/mL	<20 U/mL
Aldolase	119.8 U/L	<7.6 U/L
Myoglobin	2,220 ng/mL	28-72 ng/mL
ANA	Negative	Negative
HIV Fourth Generation	Non-reactive	Negative
Anti-Jo-1 Ab	<20 Units	<20 Units
PL-7	Negative	Negative
PL-12	Negative	Negative
EJ	Negative	Negative
OJ	Negative	Negative
SRP Autoantibodies	Negative	Negative
MI-2	Negative	Negative
TIF1-γ (P155/140)	<20 Units	<20 Units
NXP-2 (P140)	<20 Units	<20 Units
MDA-5 (P140-CADM-140)	<20 Units	<20 Units
Anti-PM/Scl-100 Ab	<20 Units	<20 Units
Fibrillarin (U3 RNP)	Negative	Negative
U2 snRNP	Negative	Negative
Anti-U1- RNP Ab	<20 Units	<20 Units
Anti-SS-A 52kD Ab, IgG	<20 Units	<20 Units
Ku	Negative	Negative

**Figure 1:**
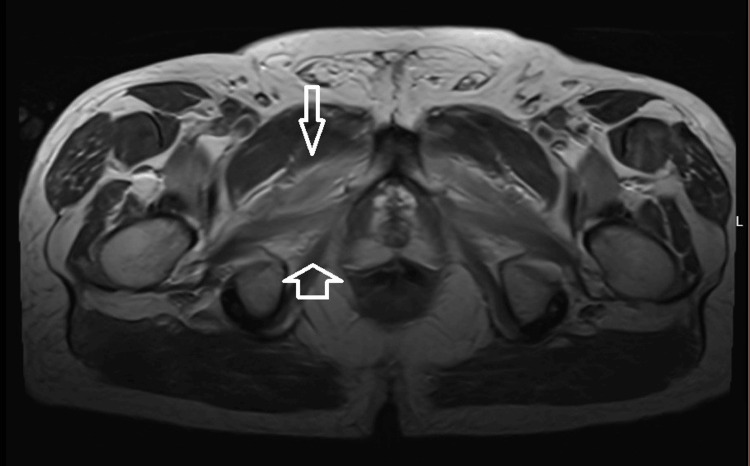
Axial T2 MRI Highlighting Symmetric Hyperintensities in the Hip Flexor Muscles and Adductor Muscles Consistent With Inflammatory Myopathy Long arrow (top): Pointing to the hip flexor muscles demonstrating the increased T2 signal consistent with myositis. Short arrow (bottom): Pointing to the adductor muscles demonstrating T2 hyperintensity.

Magnetic resonance imaging (MRI) of the bilateral thighs (non-contrast, myositis protocol) revealed T2 hyperintensities involving the adductors and hip flexors, consistent with inflammatory myopathy (Figure 1).

Muscle biopsy demonstrated scattered necrotic and regenerating fibers, consistent with IMNM. In the setting of HMGCR Ab positivity and absence of clinical or serologic features of systemic autoimmune disease (e.g., negative ANA, no rash, no arthropathy), a diagnosis of statin-associated IMNM was confirmed. High-dose oral prednisone (60 mg daily) was initiated, and the patient was discharged with appropriate prophylaxis, including trimethoprim-sulfamethoxazole for *Pneumocystis jirovecii* pneumonia (PJP) and pantoprazole for gastrointestinal protection.

**Figure 2:**
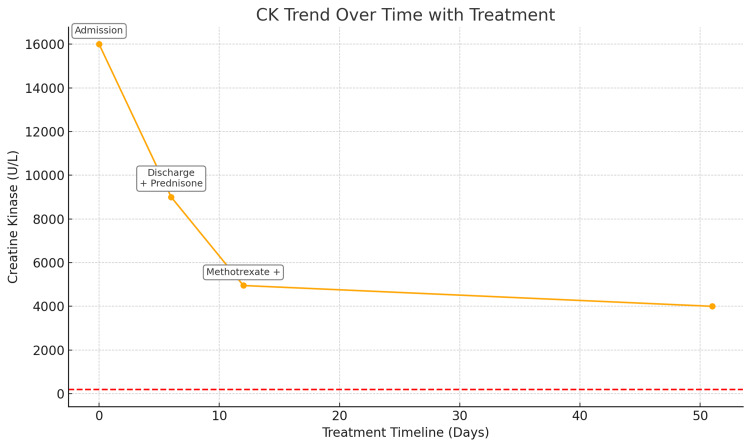
Trend of Creatine Kinase (CK) Levels in Response to Therapy and Supportive Interventions Creatine kinase (CK) trend graph demonstrating a steady decline in CK levels from admission through follow-up, in response to therapeutic interventions. The red dashed line represents the upper limit of the reference range (200 U/L). IVF = intravenous fluids; + = addition

Following discharge, the patient demonstrated a marked decline in CK levels and reported approximately 70% recovery in proximal muscle strength, with return to baseline functional capacity within several weeks (Figure 2). However, given the patient’s comorbidities, including CAD, T2DM, and HLD, prolonged corticosteroid use posed substantial cardiometabolic risk. Accordingly, methotrexate was initiated at a dose of 15 mg weekly (later increased to 20 mg) as a steroid-sparing agent, along with folic acid supplementation. He tolerated methotrexate well, without evidence of cytopenias or hepatotoxicity on serial CBC and CMP monitoring. Close collaboration with endocrinology, cardiology, rheumatology, and gastroenterology was essential to manage hyperglycemia and implement a non-statin lipid-lowering regimen, given the need for lifelong statin avoidance.

## Discussion

This case illustrates the broad clinical spectrum of statin-associated muscle toxicity, which ranges from mild, self-limiting myalgias to the rare but severe condition of NAM. While mild elevations in CK are commonly seen with statin use, NAM is a distinct immune-mediated entity marked by progressive proximal muscle weakness, markedly elevated CK levels, often exceeding 10 times the upper limit of normal, and minimal to no improvement following statin discontinuation [3,4].

The underlying pathophysiology of NAM involves autoantibody-mediated damage to muscle fibers. In particular, HMGCR, which is the pharmacologic target of statins, becomes upregulated in regenerating muscle cells. This increased expression may perpetuate antigen presentation and immune activation even after statin cessation [5].

In this patient, NAM emerged shortly after re-exposure to statin therapy following a temporary discontinuation. This sequence supports the concept of immune priming, where prior statin exposure may sensitize the immune system, leading to a more pronounced response upon reintroduction, even in patients who were asymptomatic during earlier use [1].

The patient’s clinical presentation was atypical in that he lacked myalgias, rash, or systemic inflammatory symptoms, which are more commonly associated with idiopathic inflammatory myopathies such as dermatomyositis or polymyositis [6,7]. Instead, the primary manifestation was painless, symmetric proximal weakness, and the only other laboratory clue was elevated transaminases. Liver function was otherwise unremarkable: imaging confirmed hepatic steatosis without fibrosis or cirrhosis, and serologic workup for viral, autoimmune, and metabolic liver diseases was negative. The transaminase elevations were thus retrospectively attributed to skeletal muscle injury. This case highlights the importance of considering skeletal muscle injury in the differential diagnosis when encountering unexplained elevations in liver enzymes, particularly AST and ALT, in patients with neuromuscular complaints. To our knowledge, this is among the first reported cases of HMGCR antibody-positive statin-associated NAM presenting without myalgia, rash, or systemic inflammatory symptoms.

The diagnosis was ultimately confirmed through a combination of serologic, radiologic, and histopathologic findings. MRI of the thighs revealed T2 hyperintensities in the adductors and hip flexors, consistent with active inflammation and muscle edema. MRI is a valuable non-invasive tool for identifying active myositis, localizing muscle biopsy sites, and even tracking treatment response over time [8]. Importantly, high-dose corticosteroids were withheld until after the biopsy was performed to preserve histologic clarity, as early immunosuppression can obscure necrosis, regeneration, and inflammatory infiltrates [5,9]. The muscle biopsy revealed necrotic and regenerating fibers, a hallmark feature of IMNM. Detection of HMGCR Ab further confirmed the diagnosis, as these antibodies are highly specific for statin-associated NAM and correlate with disease severity [10].

The differential diagnosis for proximal muscle weakness with elevated muscle enzymes is broad. Idiopathic inflammatory myopathies, such as polymyositis and dermatomyositis, often feature cutaneous findings, systemic inflammation, and positive ANA serology, none of which were observed in this case [6,7]. Viral myositis was unlikely due to the absence of recent infection and negative HIV screening [11]. Toxic myopathies from alcohol or medications were ruled out based on history and toxicology screens [12]. Endocrine and metabolic causes, including thyroid dysfunction, vitamin D deficiency, and electrolyte disturbances, were also excluded [13,14]. Inclusion body myositis, typically a slower-progressing asymmetric condition in older adults, was considered less likely due to the acute symmetric presentation [15]. There was no evidence of paraneoplastic, granulomatous, or sarcoid myopathies on imaging or lab testing [16,17].

The patient's laboratory evaluation was consistent with active muscle injury: CK was significantly elevated at 15,767 U/L, aldolase was 119.8 U/L, and serum myoglobin was 2,220 ng/mL. Negative ANA, normal immunoglobulin levels, and a modest ESR argued against systemic autoimmune disease. A strongly positive HMGCR Ab titer (160 U; reference <20 U) solidified the diagnosis.

Initial management included statin discontinuation and aggressive IV hydration to mitigate the risk of acute kidney injury, though CK levels plateaued near 11,000 U/L despite IV fluids, further supporting an immune-mediated myopathy. Following muscle biopsy, high-dose prednisone (60 mg daily) was initiated, along with trimethoprim-sulfamethoxazole for PJP prophylaxis and pantoprazole for gastrointestinal protection. 

In the outpatient setting, corticosteroid-induced hyperglycemia (200-300 mg/dL range) necessitated initiation of methotrexate at 15 mg weekly (later increased to 20 mg) as a steroid-sparing agent. This approach aligns with expert consensus, which advocates early Disease-Modifying Antirheumatic Drugs (DMARD) initiation in patients with metabolic risk factors or when prolonged corticosteroid use is anticipated [5,9,10]. Folic acid supplementation was co-prescribed to mitigate methotrexate toxicity, and the patient was advised to abstain from alcohol to prevent hepatotoxicity. He tolerated methotrexate well, without cytopenias or hepatic dysfunction, and remained under serial CBC and CMP monitoring.

Steroid-sparing therapy is particularly important in patients with comorbid diabetes, CAD, and HLD, all of which were present in our patient. The patient was closely followed by endocrinology and cardiology to manage corticosteroid-induced hyperglycemia and implement a statin-free lipid-lowering regimen. Relapse is common, occurring in up to 30% of patients, particularly during steroid tapering or transition to maintenance therapy [10]. Ongoing longitudinal monitoring of CK levels and muscle strength is critical. By outpatient follow-up (day 12), the patient’s CK had decreased to 4,952 U/L, and he reported approximately 70% improvement in strength with return to work.

Because statins must be permanently discontinued in patients with HMGCR-positive NAM, long-term lipid management poses a challenge. Alternatives such as proprotein convertase subtilisin/kexin type 9 (PCSK9) inhibitors or icosapent ethyl may be considered in patients requiring secondary prevention without triggering autoimmune reactivation [18]. Additionally, certain HLA genotypes, such as HLA-DRB1*11:01, may confer increased susceptibility to NAM and could inform future pharmacogenomic risk stratification [9].

This case highlights the importance of timing in clinical evaluation. The patient’s weakness began shortly after discontinuing statins, yet worsened following reinitiation, suggesting that the underlying autoimmune process may have been evolving subclinically and was subsequently unmasked or amplified by re-exposure. This highlights the value of early myositis-specific antibody testing and structured diagnostic pathways to expedite diagnosis and treatment [19]. It also illustrates the essential role of a multidisciplinary team, including rheumatology, endocrinology, and cardiology, in safely managing immunosuppression, optimizing comorbid conditions, and ensuring coordinated long-term care.

## Conclusions

Statin-associated NAM is a rare but serious immune-mediated condition that should be suspected in patients presenting with progressive proximal muscle weakness and markedly elevated CK levels, particularly in the context of recent statin initiation or re-exposure following a prior discontinuation. This case is notable for its atypical presentation in the absence of myalgias, rash, or systemic inflammatory symptoms. Elevated liver enzymes were initially misattributed to hepatic steatosis during outpatient workup, delaying recognition of underlying muscle injury. It also highlights the risk of reinitiating statins in patients with evolving subclinical NAM, which may lead to disease unmasking or exacerbation. Early recognition, a structured diagnostic approach-including muscle enzyme evaluation, myositis-specific autoantibody testing, MRI, and muscle biopsy-and timely initiation of therapy are essential to prevent irreversible muscle damage and functional decline.

Clinicians must maintain a high index of suspicion for NAM in any statin-treated patient presenting with unexplained weakness or transaminitis. Long-term management requires lifelong statin avoidance, selection of non-statin lipid-lowering alternatives, and coordination with rheumatology, endocrinology, cardiology, gastroenterology, and primary care to address both immunosuppressive therapy and comorbid conditions.
